# Construction of a circRNA-miRNA-mRNA Network Related to Macrophage Infiltration in Hepatocellular Carcinoma

**DOI:** 10.3389/fgene.2020.01026

**Published:** 2020-09-04

**Authors:** Peitao Zhou, Guanglei Zheng, Yalin Li, Dehua Wu, Yuhan Chen

**Affiliations:** ^1^Department of Radiation Oncology, Nanfang Hospital, Southern Medical University, Guangzhou, China; ^2^The First School of Clinical Medicine, Southern Medical University, Guangzhou, China

**Keywords:** circular RNA, regulatory network, macrophage, hepatocellular carcinoma, non-coding RNAs

## Abstract

Immune cells in the tumor microenvironment play a crucial role in regulating tumor progression. The circular RNA (circRNA) regulatory network involved in immune cell infiltration in hepatocellular carcinoma (HCC) remains largely unknown. In this study, the “estimate the proportion of immune and cancer cells” (EPIC) application is used to evaluate the fractions of immune cells, cancer-associated fibroblasts, and endothelial cells in HCC from The Cancer Genome Atlas (TCGA) and Gene Expression Omnibus (GEO) databases. Patients with a high macrophage fraction have better overall survival, and macrophage fraction is an independent prognostic factor for HCC. Next, the common differentially expressed mRNAs (DEmRNAs), miRNAs (DEmiRNAs), and circRNAs (DEcircRNAs) between paired tumor and non-tumor tissues are screened out from the TCGA and/or GEO databases. Through spearman correlation analysis, the macrophage-related DEmRNAs are identified to construct a circRNA-miRNA-mRNA regulatory network, which includes 6 DEcircRNAs, 7 DEmiRNAs, and 45 DEmRNAs. Functional enrichment analysis reveals that these DEmRNAs are mainly involved in immune-related processes. Furthermore, six hub DEmRNAs are identified to establish a hub circRNA regulatory network. Among the DEmRNAs in the network, PRC1 is identified as the most influential node. PRC1 high expression is correlated with poor prognosis and low macrophage infiltration in HCC. Taken together, we identify a certain circRNA regulatory network related to macrophage infiltration and provide novel insight into the mechanism of study and therapeutic targets for HCC.

## Introduction

Primary liver cancer (PLC) is currently the fourth leading cause of cancer-related death and the sixth most common malignant tumor worldwide (Villanueva, 2019). Hepatocellular carcinoma (HCC) is the predominant form of PLC, accounting for 75–85% of all cases (Bray et al., 2018). Despite advances in diagnosis and curative treatment, the optimal therapeutic methods are still elusive, and the prognosis for HCC remains poor due to late diagnosis. Therefore, an urgent need exists to identify sensitive and specific biomarkers for the early diagnosis and assessment of the prognosis of HCC (Yang J. D. et al., 2019).

The liver is recognized as a central immunological organ in the human body and is continually exposed to large numbers of circulating antigens and endotoxins from the gastrointestinal tract (Heymann and Tacke, 2016). Hepatic macrophages are essential for the maintenance of homeostasis in the liver. In the liver, macrophages are mainly derived from self-renewing tissue-resident phagocytes called Kupffer cells or circulating bone marrow-derived monocytes (Dou et al., 2020). HCC is an inflammation-related malignant tumor, and hepatic macrophages play a key role in initiation and progression of HCC (Tian et al., 2019). Macrophages are classified into two major functional phenotypes, M1 (pro-inflammatory) and M2 macrophages (anti-inflammatory). Hepatic macrophages exert a bidirectional role in the development of HCC, not only inhibiting cancer progression, but also promoting tumorigenesis via M1 and M2 macrophages, respectively. And hepatic macrophages may emerge as a prognostic factor for HCC patients (Li et al., 2009).

Circular RNA (circRNA) is a class of covalently closed single-stranded circRNA molecules formed by back-splicing. CircRNA is abundant in eukaryotic cells, structurally stable, and highly conserved with confirmation of tissue-, developmental stage-, and disease-specificity (Jeck et al., 2013). Researchers have found that circRNA can exert multiple functions, including acting as a microRNA (miRNA) sponge to modulate the miRNA-mRNA regulatory axis, directly binding to RNA-binding proteins and even translating proteins (Lei et al., 2018). Notably, circRNA plays an important role in cancer through the circRNA-miRNA-mRNA regulatory axis and thereby affects the initiation and progression of cancer (Kristensen et al., 2018). However, whether certain circRNAs can serve as an miRNA sponge to affect the activity of hepatic macrophages has not yet been reported.

The flow chart of this study design is shown in Supplementary Figure S1. Briefly, we applied the Estimate the Proportion of Immune and Cancer cells (EPIC) method (Racle et al., 2017) to estimate the infiltrating immune cells, cancer-associated fibroblasts (CAFs) and endothelial cells in HCC patients. We suggest that macrophage fraction is an independent prognostic factor for HCC from two independent data sets. Next, we analyzed the macrophage-related differentially expressed mRNAs (DEmRNAs) through correlation analysis and constructed the circRNA-miRNA-mRNA regulatory network based on these DEmRNAs. Moreover, six hub DEmRNAs were identified to establish a hub circRNA regulatory network. Among the DEmRNAs in the network, PRC1 was identified as the most influential node. High expression of PRC1 was correlated with poor prognosis and low macrophage infiltration of HCC in both data sets. These findings indicate that certain circRNA regulatory networks are involved in the process of macrophage infiltration and provide clues for a mechanism of study and therapeutic strategy development for HCC.

## Materials and Methods

### Data Collection

Three hundred and eighty-three cases (including 50 paired tumor and non-tumor samples) of transcriptome data, 98 cases (including 49 paired tumor and non-tumor samples) of miRNA-sequencing (miRNA-seq) data, and corresponding clinical information of HCC cases with overall survival (OS) time more than 30 days were downloaded from The Cancer Genome Atlas (TCGA) data portal^1^. Four hundred and thirty-five cases (including 214 paired tumor and non-tumor samples) of mRNA microarray data and corresponding clinical information on HCC cases with OS time more than 1 month were obtained from GSE14520 in the Gene Expression Omnibus (GEO) database^2^. The circRNA microarray data were retrieved from GSE94508 (five pairs of HCC and non-tumor tissues), GSE97332 (seven pairs of HCC and non-tumor tissues), and GSE78520 (three pairs of HCC and non-tumor tissues) in the GEO database. In this study, we mainly focus on the commonly differentially expressed RNAs (DERNAs) between HCC and paired non-tumor tissues, attempting to explore the consistently altered RNAs. Therefore, we did not specify the etiology of HCC cases. The TCGA transcriptome data were preprocessed by RNA-sequencing by the expectation-maximization-processed transcript per million measure. The raw data of the microarray data sets were preprocessed via background correction and normalization.

### Immune Cell Fractions and Survival Analysis

Unlike CIBERSORT or xCELL algorithms, the EPIC application could generate an absolute score that represents a cell fraction (Sturm et al., 2019). Six cell fractions, including four immune cells (B cells, CD4 T cells, CD8 T cells, and macrophages) as well as CAFs and endothelial cells of HCC were calculated by EPIC application, a public resource at https://gfellerlab.shinyapps.io/EPIC_1-1/. The association between cell fractions and survival was assessed by Kaplan–Meier survival analysis and the log-rank test. Additionally, univariate and multivariate survival analyses were performed to determine prognostic cell fractions in HCC patients.

### Screening out DERNAs

Fifty paired tumor and non-tumor samples from TCGA and 214 paired tumor and non-tumor samples from GSE14520 were used to screen out DEmRNAs. Due to the limited number of miRNA probes detected on most microarrays, we used TCGA miRNA-seq data for study. Forty-nine paired tumor and non-tumor samples from TCGA were used to screen out differentially expressed miRNA (DEmiRNAs). The DEmRNAs and DEmiRNAs between tumor and non-tumor cases were identified by R package “Bioconductor Limma.” The adjusted *P-*value (false discovery rate, FDR) of each gene was calculated by the Benjamini–Hochberg method. FDR < 0.05 and |log2FC| > 1 were used as the threshold for DEmRNA and DEmiRNA selection. Finally, the DEmRNAs and DEmiRNAs were visualized by the volcano plot and fold-change (FC) filtering. The differentially expressed circRNAs (DEcircRNAs) between tumor and non-tumor cases from multiple studies were identified by the robust rank aggregation method according to the significance score <0.01 and |log2FC| > 2 (Kolde et al., 2012). The DEcircRNAs were visualized by a heat map.

### Construction of the Macrophage-Related circRNA-miRNA-mRNA Network

The macrophage-related DEmRNAs were evaluated by the Spearman correlation analysis of the DEmRNAs and macrophage fraction. The Spearman correlation coefficient *R* > 0.15 and *P* < 0.05 were used as screening thresholds. Next, the DEcircRNA-targeted miRNAs were predicted by circRNA interactome^3^ using the context score percentile >75. The DEmiRNA-targeted genes were predicted by the microRNA data integration portal (miRDIP), which integrates more than 20 miRNA-related databases for miRNA target prediction (Tokar et al., 2018). Among the macrophage-related DEmRNAs, the potential targets of DEmiRNAs were selected with a very high score (top 1%) in miRDIP. As the sponge of miRNAs, the expression level of circRNAs usually does not affect the expression of miRNAs. In addition, some miRNAs may suppress highly expressed mRNAs in a compensatory elevated expression manner (Chen et al., 2020). Thus, circRNA-miRNA and miRNA-mRNA pairs were selected without the constraint that their expression patterns must be different. Finally, the circRNA-miRNA-mRNA regulatory network was constructed after taking the intersection of circRNA-miRNA pairs and miRNA-mRNA pairs. The regulatory network was visualized using Cytoscape 3.4.0^4^.

### Functional Enrichment and Subnetwork Analysis

Gene ontology (GO) and pathway analysis of DEmRNAs were carried out by Metascape^5^ (Zhou et al., 2019). A GO term or pathway with a *P-*value < 0.05 was considered statistically significant. The significantly enriched GO terms and pathways of DE mRNAs were ranked by −log10 (*P-*value). The potential interactions between DEmRNAs were predicted by the Search Tool for the Retrieval of Interacting Genes/Proteins (STRING) database^6^. The degree, closeness, and subnetwork analysis of the DEmRNAs were calculated by cytohubba and MCODE plugin of cytoscape. The most influential nodes within the network were evaluated by the integrated value of influence (IVI) method. The IVI method is a novel influential node-detection method that improves the performance of current tools and accurately detects influential nodes (Salavaty et al., 2020).

### Statistical Analysis

The median expression level of hub genes (DTL, EZH2, ITGA6, KIF4A, LAMC1, and PRC1) was used as the cutoff value. Based on the cutoff value, HCC patients from TCGA or GSE14520 were divided into high- and low-expression groups. OS differences between high- and low-expression groups were evaluated by Kaplan–Meier survival analysis and log-rank tests. The macrophage fraction levels between high- and low-expression groups were evaluated by the Mann–Whitney–Wilcoxon test. All tests were analyzed using R software version 3.4.2, and *P* < 0.05 was considered statistically significant.

## Results

### High Macrophage Fraction Correlates With Good Prognosis for HCC

**FIGURE 1:**
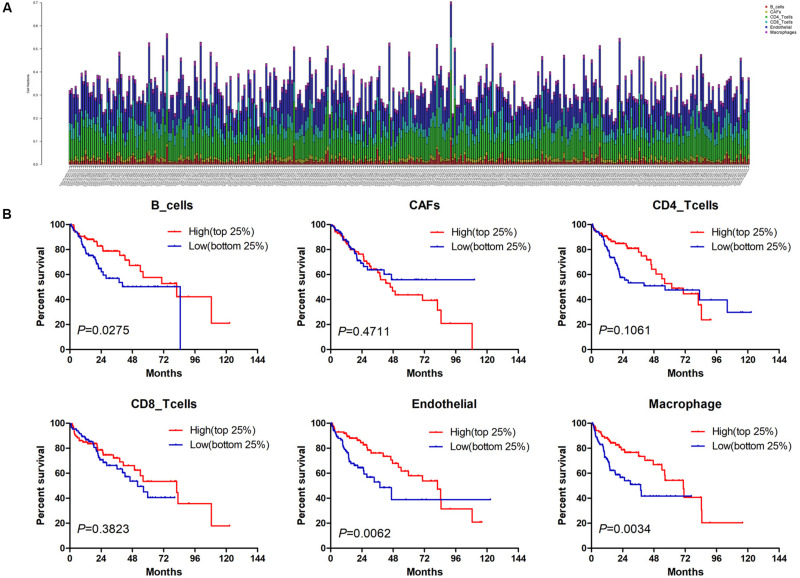
The prognostic value of six cell fractions in HCC patients from TCGA. **(A)** The composition of six cell fractions in HCC patients (*n* = 343). **(B)** The OS analysis of HCC patients with top (*n* = 86) and bottom 25% (*n* = 85) infiltration level of six cell fractions.

**FIGURE 2:**
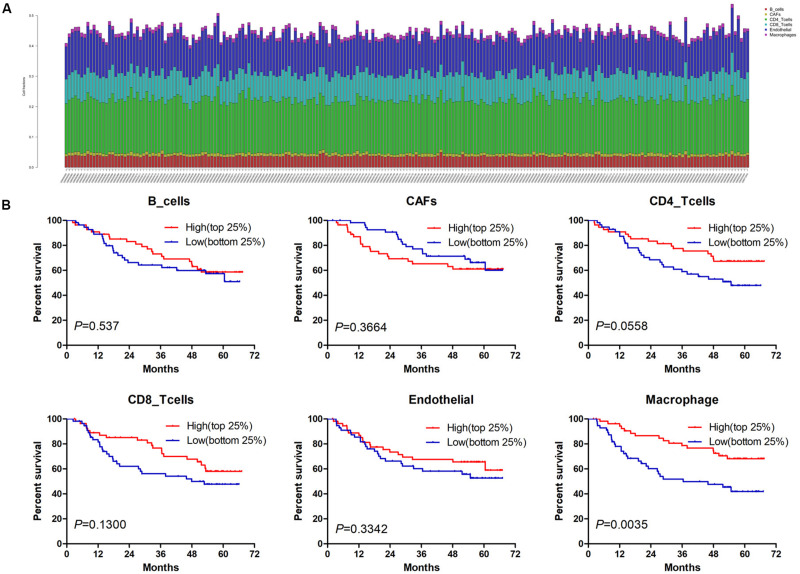
The prognostic value of six cell fractions in HCC patients from GSE14520. **(A)** The composition of six cell fractions in HCC patients (*n* = 221). **(B)** The OS analysis of HCC patients with top (*n* = 55) and bottom 25% (*n* = 56) infiltration levels of six cell fractions.

Estimate the Proportion of Immune and Cancer cells, a computer-based tool for cell fraction analysis in a tumor, can evaluate fractions of immune cells (B cells, CD4 T cells, CD8 T cells, macrophages, and NK cells), CAFs, and endothelial cells. Here, we applied EPIC to evaluate the infiltrations of cell types in TCGA HCC patients. Because the fraction of NK cells is zero in most patients, we excluded NK cells from our analysis. There existed differences in the compositions of six cell fractions between HCC patients (Figure 1A). In order to better clarify the impacts of these cell fraction differences on the survival of HCC patients, we selected the top and bottom 25% of each cell fraction for prognostic analysis. The Kaplan–Meier survival curves show that patients with high B cell, endothelial cell, and macrophage fractions had better OS than patients with lower corresponding cell fractions (Figure 1B). In addition, we used the data of HCC patients from GSE14520 for verification. The compositions of six cell fractions were different between HCC patients (Figure 2A). The prognostic analysis of each cell fraction also shows that patients with a high macrophage fraction had a good prognosis (Figure 2B). Furthermore, the multivariate Cox regression analysis shows that macrophage fraction was the independent prognostic factor for HCC patients in both TCGA and GSE14520 data sets (Tables 1, 2). These results suggest that high macrophage fraction is associated with a good prognosis for HCC patients, and macrophage infiltration may play an important role in suppressing the progression of HCC.

**TABLE 1:** Univariate and multivariate Cox regression analysis of cell fractions in HCC patients from TCGA.

Parameters	Overall survival					
	Univariate			Multivariate		
	HR	95% CI	*P*-value	HR	95% CI	*P*-value
B cells	0.85	0.72–1	0.056			
CAFs	1.08	0.78–1.48	0.646			
CD4_Tcells	0.94	0.89–0.99	0.029	0.96	0.91–1.02	0.192
CD8_Tcells	0.98	0.91–1.05	0.503			
Endothelial	0.93	0.88–0.98	0.007	0.96	0.91–1.02	0.169
Macrophages	0.36	0.18–0.72	0.004	0.46	0.22–0.97	0.041

**TABLE 2:** Univariate and multivariate Cox regression analysis of cell fractions in HCC patients from GSE14520.

Parameters	Overall Survival					
	Univariate			Multivariate		
	HR	95% CI	*P*-value	HR	95% CI	*P*-value
B cells	0.56	0.24–1.29	0.174			
CAFs	1.44	0.38–5.49	0.594			
CD4_Tcells	0.81	0.66–1	0.049	0.88	0.72–1.09	0.239
CD8_Tcells	0.74	0.5–1.07	0.112			
Endothelial	0.91	0.74–1.12	0.382			
Macrophages	0.04	0.01–0.21	<0.001	0.05	0.01–0.3	0.001

### Screening Out DEmRNAs Associated With Macrophages

**FIGURE 3:**
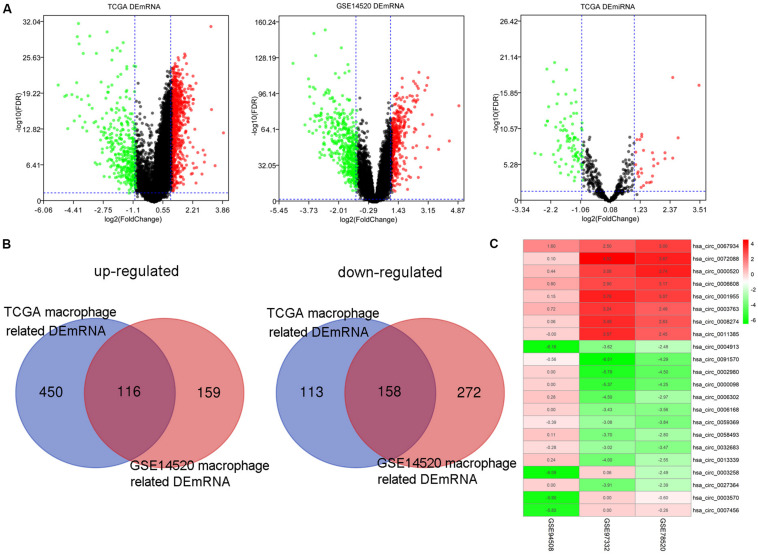
The DEmRNAs, DEmiRNAs, and DEcircRNAs identified from paired tumor and adjacent non-tumor cases. **(A)** The DEmRNAs were screened out from TCGA and GSE14520. The DEmiRNAs were screened out from TCGA. **(B)** Identification of DEmRNA-related macrophages from TCGA and GSE14520. **(C)** The DEcircRNAs were screened out from three GEO data sets.

One thousand, one hundred eighty DEmRNAs (794 up and 386 down) were screened out from 50 paired cancer and adjacent tissues of TCGA HCC patients (Figure 3A). Eight hundred thirty-seven DEmRNAs (566 up and 271 down) were found to associate with macrophages by Spearman correlation analysis. Eight hundred thirty-one DEmRNAs (356 up and 475 down) were identified from 214 matched cancer and adjacent tissues of GSE14520 HCC patients (Figure 3A). Spearman correlation analysis revealed 705 DEmRNAs (275 up and 430 down) related to macrophages. Finally, 274 DEmRNAs (116 up and 158 down) associated with macrophages were obtained after taking the intersection of the results from TCGA and GSE14520 (Figure 3B). Enrichment analysis of these DEmRNAs shows that these genes could be enriched in immune-related processes, such as acute inflammatory response, initial triggering of complement, response to tumor necrosis factor, positive chemotaxis, positive regulation of adaptive immune response, and negative regulation of defense response (Supplementary Figure S2).

### Construction of the circRNA-miRNA-mRNA Regulatory Network Related to Macrophages

**FIGURE 4:**
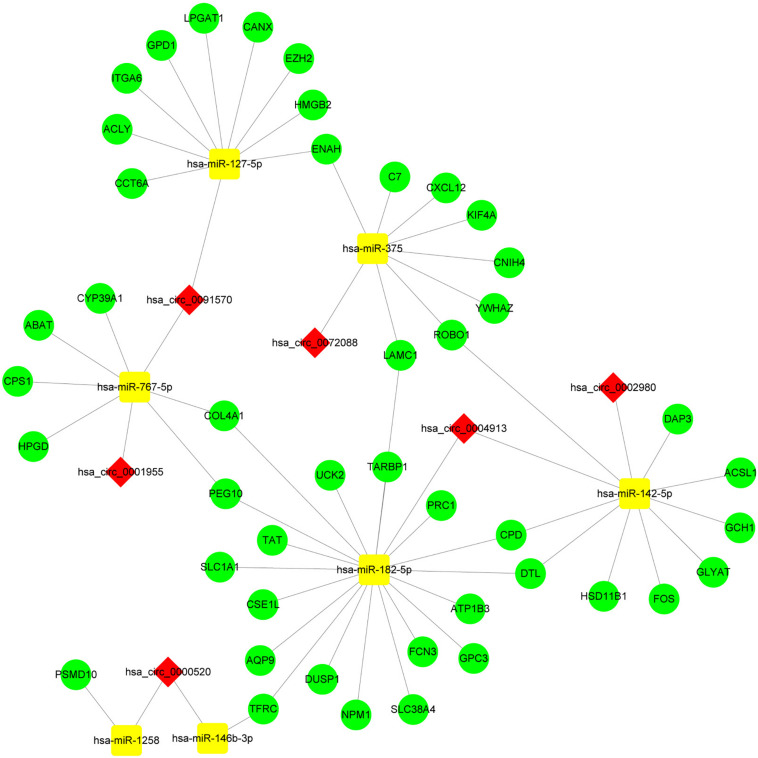
Construction of the circRNA-miRNA-mRNA network based on 45 DEmRNAs related to macrophages. The network was constructed after taking the intersection of DEcircRNA-DEmiRNA pairs and DEmiRNA-macrophage-related DEmRNA pairs. The red diamond, yellow rectangle, and light green ellipse indicate circRNA, miRNA, and mRNA, respectively.

By screening miRNA-seq data from paired tumor and adjacent non-tumor tissues in TCGA HCC cases, a total of 121 DEmiRNAs (29 up and 92 down) were obtained (Figure 3A). From the circRNA microarray data of paired tumor and adjacent non-tumor tissues in three GEO data sets, a total of 22 DEcircRNAs (8 up and 14 down) were identified (Figure 3C). Two hundred ninety-two circRNA-miRNA pairs and 4820 miRNA-mRNA pairs were predicted by circinteractome and miRDIP, respectively. After taking the intersection of these RNA pairs and DEmRNAs coexpressed with macrophages, 45 DEmRNAs, 7 DEmiRNAs, and 6 DEcircRNAs were utilized to construct a macrophage-related circRNA-miRNA-mRNA regulatory network. This network contains 9 circRNA-miRNA pairs and 52 miRNA-mRNA pairs (Figure 4).

### Construction of the Hub circRNA-miRNA-mRNA Regulatory Network Related to Macrophages

**FIGURE 5:**
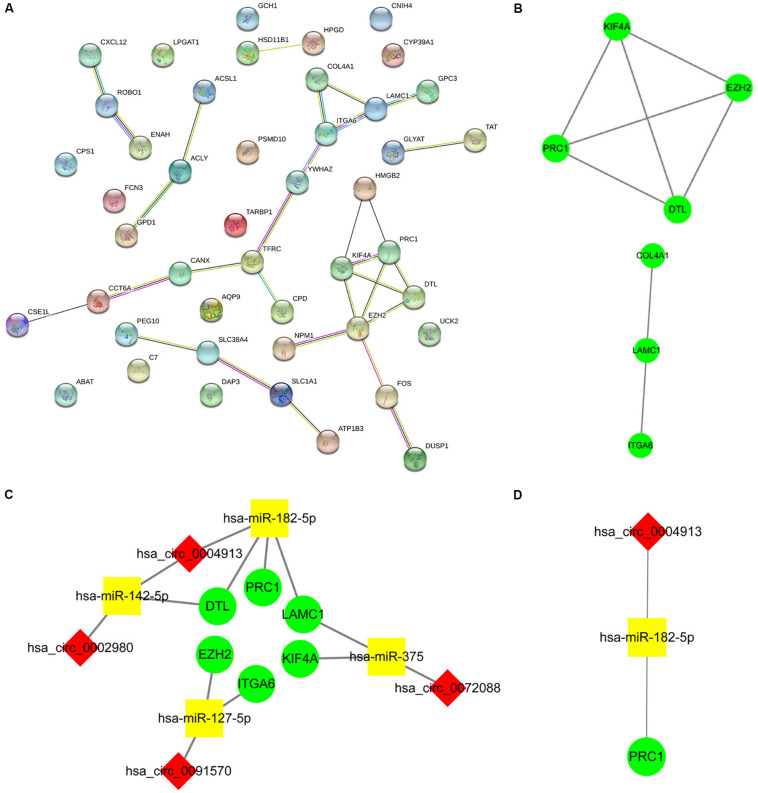
Construction of the hub circRNA-miRNA-mRNA network. **(A)** PPI network analysis of 45 DEmRNAs related to macrophage. **(B)** Two subnetworks of PPI network analyzed by the MCODE plugin of cytoscape. **(C)** Construction of the circRNA-miRNA-mRNA network based on six hub DEmRNAs related to macrophages. **(D)** Construction of the circRNA-miRNA-mRNA network based on PRC1. PRC1 was identified as the most influential node by the IVI method. The red diamond, yellow rectangle, and light green ellipse indicate circRNA, miRNA, and mRNA, respectively.

Enrichment analysis of 45 macrophage-related DEmRNAs reveals that these genes could be enriched in immune-related processes, such as response to lipopolysaccharide, cytokine-mediated signaling pathway, and antigen processing and presentation of exogenous peptide antigen (Supplementary Figure S3). The potential interactions of 45 DEmRNAs were analyzed on the STRING database (Figure 5A). The degree and closeness of the DEmRNAs were calculated by the cytohubba plugin of cytoscape (Supplementary Table 1). Two subnetworks of DEmRNAs were obtained through the MCODE plugin of cytoscape (Figure 5B). Based on the top 10 DEmRNAs ranked by degree and closeness as well as seven DEmRNAs enriched in two subnetworks, six hub DEmRNAs, namely enhancer of zeste homolog 2 (EZH2), kinesin family member 4A (KIF4A), polycomb repressive complex 1 (PRC1), integrin alpha 6 (ITGA6), denticleless E3 ubiquitin protein ligase homolog (DTL), and Laminin C1 (LAMC1), were finally screened out. A hub circRNA-miRNA-mRNA regulatory network related to macrophages was constructed based on these six hub DEmRNAs (Figure 5C). Among this hub network, all DEmRNAs were upregulated. Hsa-miR-182-5p was up-regulated while hsa-miR-142-5p and hsa-miR-375 were down-regulated. Four DEcircRNAs, except hsa_circ_0072088, were downregulated in HCC. We further analyzed the expression of 6 hub DEmRNAs in different GEO data sets of HCC by using HCCDB, a database of the HCC expression atlas (Lian et al., 2018). As shown in Supplementary Figure S4 and Supplementary Tables 2, six hub DEmRNAs were upregulated in most GEO data sets or the International Cancer Genome Consortium (ICGC) project, which is consistent with the results from TCGA. For hub miRNAs, all of them, except hsa-miR-127-5p, were significantly dysregulated in HCC from some GEO data sets analyzed by dbDEMC 2.0 (Supplementary Table 3), a database of differentially expressed miRNAs in human cancers (Yang et al., 2017). The expression status of hsa-miR-182-5p, hsa-miR-142-5p, and hsa-miR-375 in HCC were in line with the TCGA results.

### High Expression of DTL or PRC1 Is Associated With Poor Prognosis and Low Macrophage Infiltration of HCC

**FIGURE 6:**
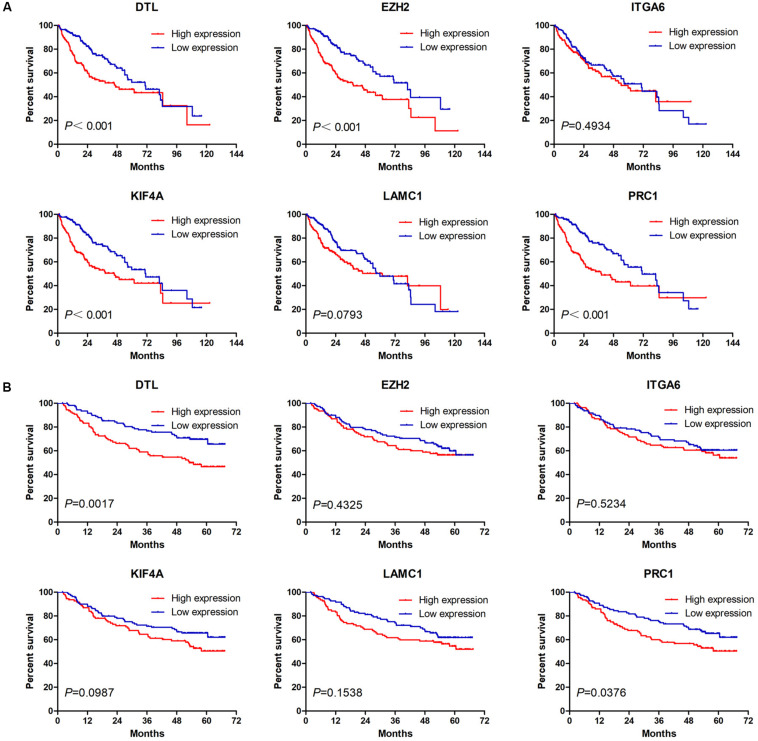
The Kaplan–Meier survival analysis of six hub macrophage-related DEmRNAs in HCC cases from TCGA **(A)** and GSE14520 **(B)**.

**FIGURE 7:**
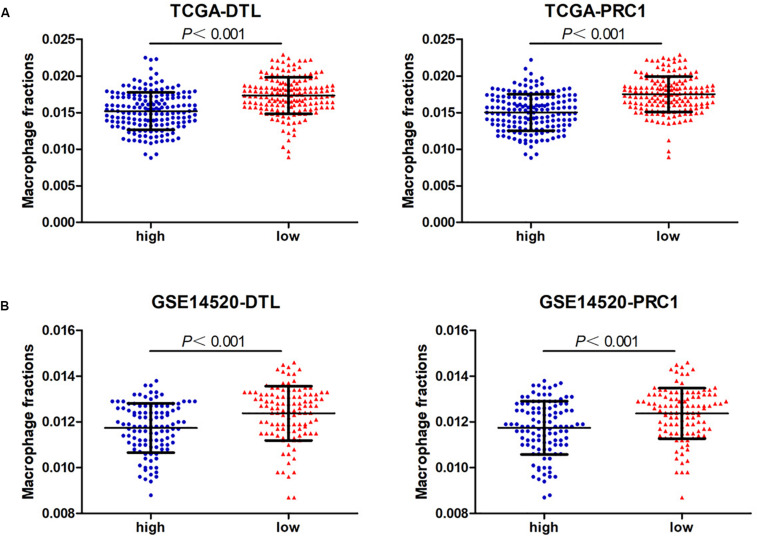
The macrophage fractions in HCC cases with different expression levels of PRC1 or DTL from TCGA **(A)** and GSE14520 **(B)**.

Next, we analyzed the prognostic value of these six hub DEmRNAs. High expression of DTL, EZH2, KIF4A, or PRC1 was associated with poor prognosis of HCC patients from TCGA (Figure 6A). In GSE14520, HCC patients with high DTL or PRC1 expression had shorter OS (Figure 6B). Data from both TCGA and GSE14520 show that high expression of DTL or PRC1 was associated with poor prognosis of patients with HCC. PRC1 or DTL expression status was also identified to have prognostic value for HCC patients in other GEO data sets or the ICGC project (Supplementary Figure 4). In addition, we further applied the IVI method to test whether PRC1 or DTL was the most influential node within the network. Those coexpressed DEmRNAs with co-expression value >0.3 calculated by STRING were selected for IVI analysis (Supplementary Table 4). According to the results, both PRC1 and KIF4A had the highest IVI values (Supplementary Table 5). However, KIF4A high expression was correlated with the poor survival of HCC patients in TCGA but not in GSE14520. Therefore, a core circRNA-miRNA-mRNA regulatory network was constructed based on PRC1 (Figure 5D). Furthermore, we analyzed the macrophage fractions of HCC cases with different PRC1 or DTL expression in TCGA and GSE14520, respectively. Patients with PRC1 or DTL high expression showed low macrophage fractions in both TCGA (Figure 7A) and GSE14520 (Figure 7B). Combined with the poor prognosis of low macrophage infiltration mentioned before, the reason for the poor prognosis of HCC patients with PRC1 or DTL high expression may be explained to some extent.

## Discussion

As important innate immune cells in the tumor immune microenvironment, macrophages play pivotal roles in the tumorigenesis and development of cancer as well as impact the efficacy of immunotherapy (Cai et al., 2019). In addition to using long non-coding RNA as the biomarker for cancers (Hajjari and Salavaty, 2015), numerous studies have confirmed that circRNA acts as an miRNA sponge to modulate the pathogenesis of cancer, which facilitates it to serve as a diagnostic and prognostic biomarker and even a therapeutic target for HCC (Fu et al., 2018). In the present study, we demonstrate that macrophage infiltration is an independent prognostic factor for HCC. Based on identified macrophage-related DEmRNAs, a circRNA-miRNA-mRNA regulatory network was constructed. We also constructed a hub circRNA–miRNA-mRNA network, containing six hub DEmRNAs, among which two hub DEmRNAs were found to be associated with poor prognosis and low macrophage infiltration of HCC.

We used the EPIC tool to identify the proportion of six cell types in HCC patients in both the TCGA and GEO data sets and found that patients with a high macrophage fraction had better OS. Hepatic macrophages, derived from resident Kupffer cells and recruited circulating monocytes, are considered to be a double-edged sword in the pathogenesis of HCC (Weston et al., 2019). Hepatic macrophage in HCC is a highly heterogeneous and plastic population, containing both tumor-suppressive (M1 macrophages) and tumor-promotive (M2 macrophages) subtypes, which are related to different prognoses in HCC patients (Tian et al., 2019; Dou et al., 2020). Rohr-Udilova et al. (2018) show that an increase in M1 macrophages may contribute to the favorable prognosis of HCC patients. Further studies demonstrate that CD68 + CD169 + macrophages and CD68+CD38+macrophages are associated with better survival while CD68+CD163+ (M2 macrophages) and total CD68+ macrophages indicate a worse survival in patients with HCC (Li et al., 2017; Lam et al., 2019). Using single-cell analysis, MacParland et al. (2018) classified Kupffer cells into two populations, CD68+MARCO− (macrophage receptor with collagenous structure) and CD68+MARCO+ macrophages. High intratumoral MARCO expression correlates with favorable prognosis of HCC while downregulation of MARCO promotes tumor cell migration and invasion, resulting in tumor progression (Sun et al., 2017). Our analysis indicates that high macrophage fraction had better OS in HCC patients from TCGA and GEO data sets. Combining our results with previous evidence, we carefully assume that HCC patients with a high macrophage fraction show better OS, which is likely attributed to the fact that M1 macrophages may be the dominant tumor-infiltrating macrophages. However, in EPIC, macrophages have not been classified into different polarization status for further analysis. It may be interesting and valuable to investigate the polarization status of macrophages in HCC in future study.

In order to further demonstrate the RNA regulatory network related to macrophage infiltration in HCC, we screened out the macrophage-related DEmRNAs between tumor and non-tumor tissues and then constructed the circRNA-miRNA-mRNA network. Finally, six DEcircRNAs were included in the regulatory network. Among them, previous studies confirm that hsa_circ_0072088 (circZFR) and hsa_circ_0091570 could function as miRNA sponges to affect the progression of HCC (Tan et al., 2019; Wang et al., 2019; Yang X. et al., 2019). However, to date, none of the six DEcircRNAs have been reported to modulate macrophage functions related to HCC. Further studies should focus on the mechanism in which circRNA serves as an miRNA sponge to modulate the macrophage activity to affect the tumor microenvironment of HCC.

In this study, we identified seven DEmiRNAs in the regulatory network. Previous studies have demonstrated the important role of some miRNAs in modulating functions of macrophages in the liver. It is reported that miR-142-5p could regulate macrophage profibrogenic gene expression to promote liver fibrosis (Su et al., 2015). Additionally, miR-182-5p inhibits proinflammatory cytokine production from LPS-induced macrophages to attenuate liver ischemia-reperfusion injury (Jiang et al., 2016). Moreover, miR-375 could reduce Kupffer cell apoptosis to improve immune function during liver failure (Ke et al., 2019). There is no report about these miRNAs being involved in the regulation of macrophage infiltration related to the pathogenesis of HCC. However, most of these miRNAs have been reported to play crucial roles in the development of HCC (Lou et al., 2017; Cao et al., 2018). Combination with the miRNA regulatory effect in modulating macrophage infiltration and activity may be a promising direction for future mechanism studies of HCC.

To further identify the key circRNAs involved in the regulatory network, we performed PPI network analysis, and six hub DEmRNAs (EZH2, DTL, PRC1, KIF4A, ITGA6, and LAMC1) were identified for the construction of the hub circRNA regulatory network, including nine circRNA-miRNA-mRNA axes. Among the six hub DEmRNAs, EZH2 has been demonstrated to be highly expressed in HCC, and injection of EZH2 inhibitor GSK126 induces CXCL10 production from macrophages and causes plasma cell polarization, inhibition of the antitumor T cell response, and hepatoma growth (Wei et al., 2019). Additionally, inhibition of EZH2 is also able to affect the macrophage activity in some other cancer types, such as suppressing macrophage infiltration in lung cancer and shifting microglia toward the M1 phenotype in glioblastoma (Yin et al., 2017; Xia et al., 2019). Although the role of DTL, PRC1, KIF4A, ITGA6, and LAMC1 in the regulation of HCC-related macrophages remains unknown, the findings of the mentioned hub DEmRNAs in the regulation of macrophage activity in other diseases may provide the clues for HCC. For instance, PRC1, a microtubule binding and bundling protein, is pivotal in maintaining the mitotic spindle midzone. PRC1 promotes tumor cell self-renewal and induces the recruitment of M2-like tumor-associated macrophages and regulatory T cells, leading to metastasis initiation of double-negative prostate cancer (Su et al., 2019). In addition, PRC1 binds to the motor protein KIF4A to form central spindles and the midzone properly, which is essential for cell-cycle progression and cytokinesis (Wijeratne and Subramanian, 2018). Increasing KIF4A can promote the recruitment of macrophages toward oral squamous cell carcinoma cells and educate them to M2 polarized macrophages through regulating CCL2/CCR2 (Zhang et al., 2017). Based on these reports, we speculate that some identified DEmRNAs in HCC cells may affect macrophage infiltration through releasing certain cytokines.

Among the network, PRC1 was identified as the most influential node by the IVI method. High expression of PRC1 was associated with poor prognosis and low macrophage infiltration in HCC, indicating PRC1 may play an important role in regulating macrophage infiltration into HCC tissues. Therefore, we constructed a core circRNA-miRNA-mRNA regulatory axis based on PRC1, namely hsa_circ_0004913/hsa-miR-182-5p/PRC1. Based on the effect of PRC1 on macrophages (Su et al., 2019), we hypothesize that hsa_circ_0004913 acts as the hsa-miR-182-5p sponge to relieve PRC1 inhibition, leading to a decrease in M1 macrophages and an increase in M2 macrophage infiltration, thereby promoting the progression of HCC.

## Conclusion

In conclusion, we suggest that macrophage fraction serves as an independent prognostic factor for HCC. Next, we identified the macrophage-related DEmRNAs and constructed the circRNA-miRNA-mRNA regulatory network based on these DEmRNAs. Moreover, six hub DEmRNAs were identified to establish a hub circRNA regulatory network through PPI analysis. PRC1, the most influential node among the network, was associated with poor prognosis and low macrophage infiltration of HCC. However, there are some limitations in this study. First, we did not specify the etiology of the HCC cases. These identified RNAs may be common rather than representative in HCC with different etiologies. To gain insight into the etiology-associated circRNA regulatory networks in HCC is another focus in our future study. Second, the sample size of HCC tissues with circRNA data was small. There are only 15 pairs of tumor and non-tumor samples although we have included three GEO data sets for study. Moreover, most of the results are based solely on bioinformatics models, and other studies are needed to verify our hypothesis. Taken together, our findings indicate that certain circRNA regulatory networks are involved in the process of macrophage infiltration and provide a novel insight into the mechanism of study and therapeutic targets for HCC.

## Data Availability Statement

Publicly available datasets were analyzed in this study. The data can be found here: TCGA; GSE14520; GSE94508; GSE97332; GSE78520.

## Author Contributions

YC and DW designed the study. PZ performed the specific procedures and wrote the manuscript. GZ and YL analyzed the data and made the pictures and graphs. All authors have read and approved the manuscript.

## Conflict of Interest

The authors declare that the research was conducted in the absence of any commercial or financial relationships that could be construed as a potential conflict of interest.

## Footnotes

1 https://tcga-data.nci.nih.gov/tcga/

2 http://www.ncbi.nlm.nih.gov/gds/

3 https://circinteractome.nia.nih.gov/

4 http://cytoscape.org/

5 https://metascape.org/gp/index

6 https://stringdb.org/
