# Supplementary material for: Construction of a circRNA-miRNA-mRNA Network Related to Macrophage Infiltration in Hepatocellular Carcinoma
Source: Front Genet. 2020 Sep 4;11:1026. doi: 10.3389/fgene.2020.01026 (PMC7500212; doi:10.3389/fgene.2020.01026)
Supplement: Supplementary file 1 [file Data_Sheet_1.docx]

Supplementary Material

# Supplementary Figures and Tables

## Supplementary Figures

FIGURE S1 Flow chart of the study design.

FIGURE S2 Functional enrichment analysis of 274 DEmRNAs related to macrophage by Metascape.

FIGURE S3 Functional enrichment analysis of 45 DEmRNAs related to macrophage by Metascape.

FIGURE S4 The expression status and prognostic value of 6 hub DEmRNAs in different GEO, TCGA, ICGC or GTEx data analyzed by HCCDB. Diff: the number of differentially expressed datasets; Red/Blue for consensus up-regulated/down-regulated. Prognosis: the number of significant datasets by survival analysis; Red/Blue for UNFavorable/Favorable. HCC/AllTumor: Red/Blue for positive/negative fold change in log2 scale by comparing HCC with all tumors (TCGA data). HCC/AllAdjacent: Red/Blue for positive/negative fold change in log2 scale by comparing HCC with all adjacent samples (TCGA data). HCC/Adjacent: Red/Blue for positive/negative fold change in log2 scale by comparing HCC with adjacent samples (HCCDB data). Liver/OtherNormal: Red/Blue for positive/negative fold change in log2 scale by comparing liver with normal tissues (GTEx&TCGA data). Detail information could be retrieved from HCCDB (http://lifeome.net/database/hccdb/home.html).

## Supplementary Tables

Supplementary Table 1 The degree and closeness of DEmRNA in circRNA network calculated by cytohubba.

| Node name | Degree | Node name | Closeness |
| --- | --- | --- | --- |
| EZH2 | 5 | EZH2 | 6 |
| KIF4A | 4 | KIF4A | 5.33333 |
| PRC1 | 4 | PRC1 | 5.33333 |
| DTL | 3 | TFRC | 5.25 |
| TFRC | 3 | ITGA6 | 5.11667 |
| LAMC1 | 3 | YWHAZ | 4.91667 |
| ITGA6 | 3 | DTL | 4.83333 |
| SLC38A4 | 2 | LAMC1 | 4.7 |
| ACLY | 2 | CANX | 4.53333 |
| CCT6A | 2 | FOS | 4.33333 |

Supplementary Table 2 The expression level of 6 hub DEmRNAs in different GEO, TCGA or ICGC data analyzed by HCCDB.

| Data | Type | PRC1 | | DTL | | KIF4A | | EZH2 | | LAMC1 | | ITGA6 | |
| --- | --- | --- | --- | --- | --- | --- | --- | --- | --- | --- | --- | --- | --- |
|  |  | *P* | Mean | *P* | Mean | *P* | Mean | *P* | Mean | *P* | Mean | *P* | Mean |
| GSE22058 | HCC | 3.14E-53 | 9.253 | 2.71E-48 | 8.273 | 6.35E-47 | 8.112 | 2.01E-37 | 7.658 | 9.59E-23 | 9.45 | 7.95E-44 | 11.03 |
|  | Adjacent |  | 6.557 |  | 5.452 |  | 6.021 |  | 6.033 |  | 8.308 |  | 9.845 |
| GSE25097 | HCC | 1.05E-52 | 1.518 | 1.96E-46 | 0.8624 | 8.17E-54 | 0.7561 | 5.27E-47 | 0.6373 | 1.39E-45 | 3.495 | 5.14E-76 | 5.708 |
|  | Adjacent |  | 0.2008 |  | 0.1025 |  | 0.1418 |  | 0.1679 |  | 1.524 |  | 2.763 |
|  | Cirrhotic |  | 0.3194 |  | 0.1918 |  | 0.215 |  | 0.1656 |  | 2.487 |  | 3.901 |
|  | Healthy |  | 0.08417 |  | 0.04075 |  | 0.1138 |  | 0.1557 |  | 1.475 |  | 2.636 |
| GSE36376 | HCC | 3.53E-91 | 8.138 | 7.33E-41 | 6.857 | 1.67E-45 | 6.247 | 1.96E-43 | 6.825 | 8.82E-86 | 9.741 | 1.79E-39 | 6.228 |
|  | Adjacent |  | 6.233 |  | 6.449 |  | 5.652 |  | 6.503 |  | 7.953 |  | 5.973 |
| GSE14520 | HCC | 9.72E-89 | 6.223 | 3.87E-76 | 6.185 | 4.28E-61 | 5.022 | 1.48E-67 | 5.652 | 7.03E-68 | 7.989 | 1.63E-61 | 6.052 |
|  | Adjacent |  | 3.766 |  | 3.913 |  | 3.592 |  | 3.883 |  | 6.521 |  | 4.687 |
| GSE10143 | HCC | 0.00000184 | 12.37 |  |  |  |  | 5.91E-11 | 11.19 | 2.88E-07 | 13.15 | 3.90E-09 | 14.01 |
|  | Adjacent |  | 11.9 |  |  |  |  |  | 10.33 |  | 12.53 |  | 13.5 |
| GSE46444 | HCC | 0.04076 | 7.741 | 0.00547 | 6.189 | 0.4964 | 6.572 | 0.9986 | 6.734 | 0.8461 | 5.929 | 0.8598 | 5.549 |
|  | Adjacent |  | 8.114 |  | 6.726 |  | 6.362 |  | 6.735 |  | 5.957 |  | 5.541 |
| GSE54236 | HCC | 9.94E-17 | 9.102 | 6.25E-12 | 6.976 | 8.96E-15 | 6.233 | 3.48E-12 | 9.791 | 0.00000405 | 9.495 | 1.82E-08 | 10.09 |
|  | Adjacent |  | 7.553 |  | 5.695 |  | 4.237 |  | 8.855 |  | 8.945 |  | 9.484 |
| GSE63898 | HCC | 1.22E-15 | 4.038 | 4.87E-37 | 5.183 | 5.95E-23 | 4.878 | 7.22E-23 | 4.378 | 4.31E-42 | 7.68 | 4.34E-53 | 8.78 |
|  | Adjacent |  | 3.879 |  | 4.16 |  | 4.062 |  | 3.987 |  | 6.58 |  | 7.239 |
| TCGA-LIHC | HCC | 1.00E-32 | 8.639 | 2.57E-25 | 7.663 | 7.05E-40 | 7.53 | 5.21E-29 | 7.888 | 5.61E-34 | 11.73 | 4.22E-17 | 10.56 |
|  | Adjacent |  | 5.355 |  | 4.298 |  | 3.522 |  | 5.324 |  | 10.53 |  | 9.225 |
| GSE64041 | HCC | 2.06E-14 | 6.991 | 1.67E-11 | 7.678 | 1.19E-13 | 5.222 | 6.81E-14 | 7.612 | 6.20E-09 | 8.984 | 2.34E-13 | 9.323 |
|  | Adjacent |  | 5.715 |  | 6.29 |  | 4.616 |  | 6.615 |  | 8.346 |  | 8.346 |
| GSE76427 | HCC | 6.89E-28 | 9.32 | 4.31E-15 | 7.785 | 2.15E-17 | 7.387 | 2.25E-15 | 7.233 | 1.90E-09 | 10.97 | 0.1347 | 6.761 |
|  | Adjacent |  | 7.733 |  | 7.196 |  | 6.703 |  | 6.881 |  | 10.21 |  | 6.732 |
| ICGC-LIRI-JP | HCC | 1.11E-74 | 2.762 | 5.28E-59 | 1.593 | 7.32E-57 | 1.519 | 1.11E-65 | 2.916 | 5.86E-49 | 3.873 |  |  |
|  | Adjacent |  | 0.8453 |  | 0.3905 |  | 0.304 |  | 1.564 |  | 2.533 |  |  |

Supplementary Table 3 The expression status of 3 hub miRNAs in different GEO data analyzed by dbDEMC 2.0.

| miRNA | GEO series | logFC | adj Pvalue | Status |
| --- | --- | --- | --- | --- |
| hsa-miR-182-5p | GSE39678 | 1.63566238 | 0.01664754 | UP |
| hsa-miR-142-5p | GSE22058 | -0.23837079 | 1.06E-07 | DOWN |
| hsa-miR-142-5p | GSE21362 | -1.09006903 | 0.001303863 | DOWN |
| hsa-miR-375 | GSE22058 | -0.76351913 | 1.33E-15 | DOWN |
| hsa-miR-375 | GSE20077 | -4.833049214 | 1.84E-05 | DOWN |
| hsa-miR-375 | GSE21362 | -2.325864661 | 2.24E-12 | DOWN |
| hsa-miR-375 | GSE36915 | -1.479586375 | 0.005858103 | DOWN |

Supplementary Table 4 The coexpression value of 45 DEmRNAs related to macrophage by STRING database.

| node1 | node2 | node1_string_internal_id | node2_string_internal_id | coexpression |
| --- | --- | --- | --- | --- |
| PRC1 | KIF4A | 4446249 | 4444386 | 0.937 |
| ROBO1 | ENAH | 4449112 | 4442751 | 0.162 |
| FOS | DUSP1 | 4438204 | 4433734 | 0.873 |
| ITGA6 | YWHAZ | 4447442 | 4446480 | 0 |
| ITGA6 | LAMC1 | 4447442 | 4434618 | 0.108 |
| ROBO1 | CXCL12 | 4449112 | 4446453 | 0.065 |
| TFRC | CPD | 4442333 | 4433330 | 0 |
| GPC3 | LAMC1 | 4446259 | 4434618 | 0.076 |
| PRC1 | DTL | 4446249 | 4442781 | 0.812 |
| KIF4A | DTL | 4444386 | 4442781 | 0.776 |
| ATP1B3 | SLC1A1 | 4436673 | 4435062 | 0.062 |
| ITGA6 | COL4A1 | 4447442 | 4444632 | 0.097 |
| DTL | EZH2 | 4442781 | 4439336 | 0.725 |
| COL4A1 | LAMC1 | 4444632 | 4434618 | 0.321 |
| PRC1 | EZH2 | 4446249 | 4439336 | 0.672 |
| EZH2 | FOS | 4439336 | 4438204 | 0 |
| EZH2 | NPM1 | 4439336 | 4437352 | 0.131 |
| PRC1 | HMGB2 | 4446249 | 4437293 | 0.56 |
| HSD11B1 | HPGD | 4442791 | 4437300 | 0 |
| KIF4A | EZH2 | 4444386 | 4439336 | 0.528 |
| TFRC | CANX | 4442333 | 4434023 | 0.087 |
| CCT6A | CANX | 4436191 | 4434023 | 0.121 |
| ACSL1 | ACLY | 4449224 | 4434323 | 0.063 |
| GPD1 | ACLY | 4437724 | 4434323 | 0.064 |
| SLC38A4 | SLC1A1 | 4447632 | 4435062 | 0.064 |
| PEG10 | SLC38A4 | 4448998 | 4447632 | 0.061 |
| KIF4A | HMGB2 | 4444386 | 4437293 | 0.453 |
| YWHAZ | TFRC | 4446480 | 4442333 | 0.062 |
| GLYAT | TAT | 4451863 | 4441813 | 0.099 |
| CCT6A | CSE1L | 4436191 | 4435176 | 0.414 |

Supplementary Table 5 The Integrated Value of Influence (IVI) value of the co-expressed DEmRNAs.

| DEmRNA | IVI |
| --- | --- |
| PRC1 | 100 |
| KIF4A | 100 |
| DTL | 1.981539 |
| EZH2 | 1.981539 |
| HMGB2 | 1.500626 |
| FOS | 1 |
| COL4A1 | 1 |
| CCT6A | 1 |
| DUSP1 | 1 |
| LAMC1 | 1 |
| CSE1L | 1 |
