# Supplementary figures and images for: Construction of a circRNA-miRNA-mRNA Network Related to Macrophage Infiltration in Hepatocellular Carcinoma
Source: Front Genet. 2020 Sep 4;11:1026. doi: 10.3389/fgene.2020.01026 (PMC7500212; doi:10.3389/fgene.2020.01026)

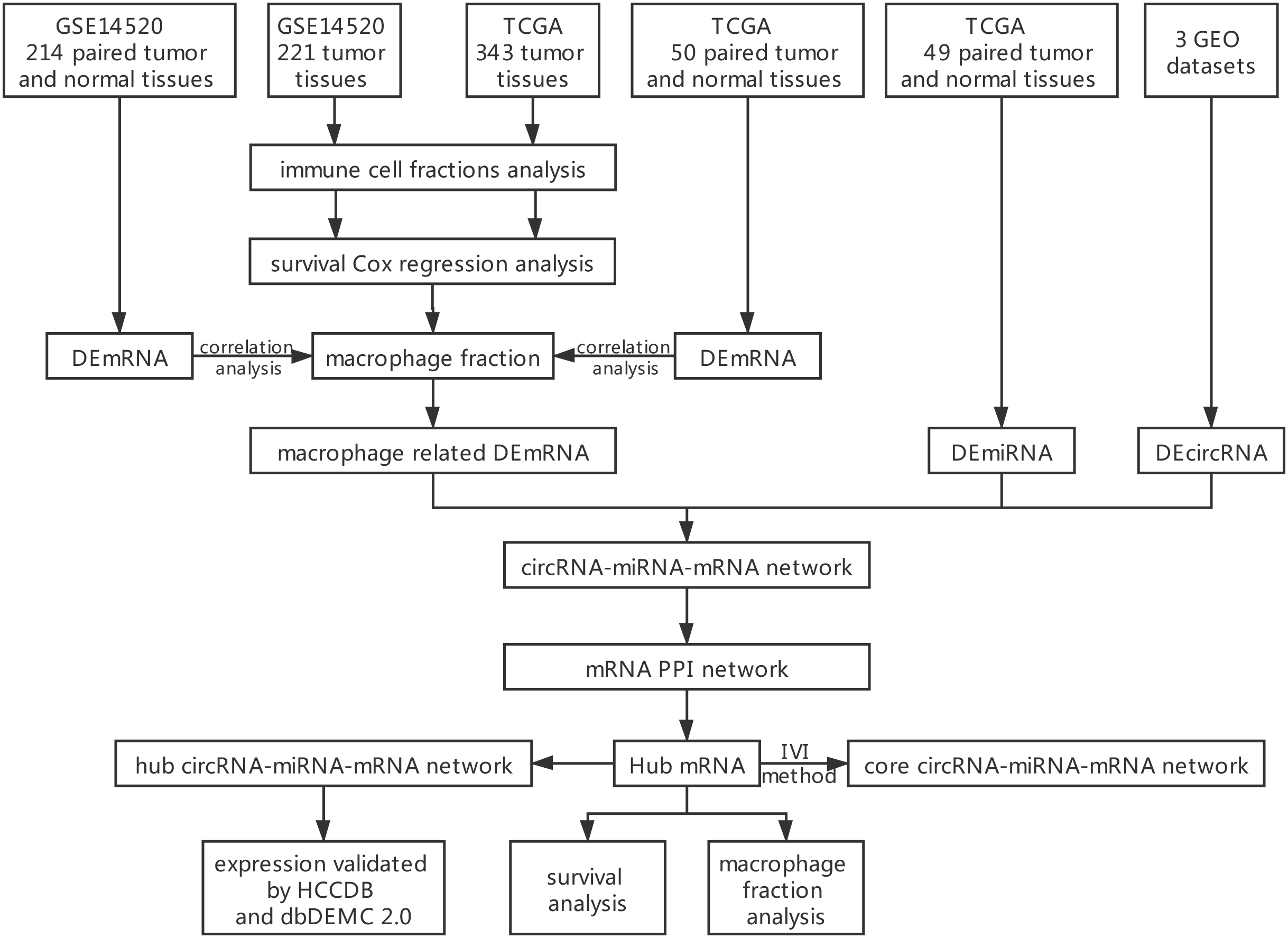

Supplement: Supplementary file 2 [file Image_1.JPEG]

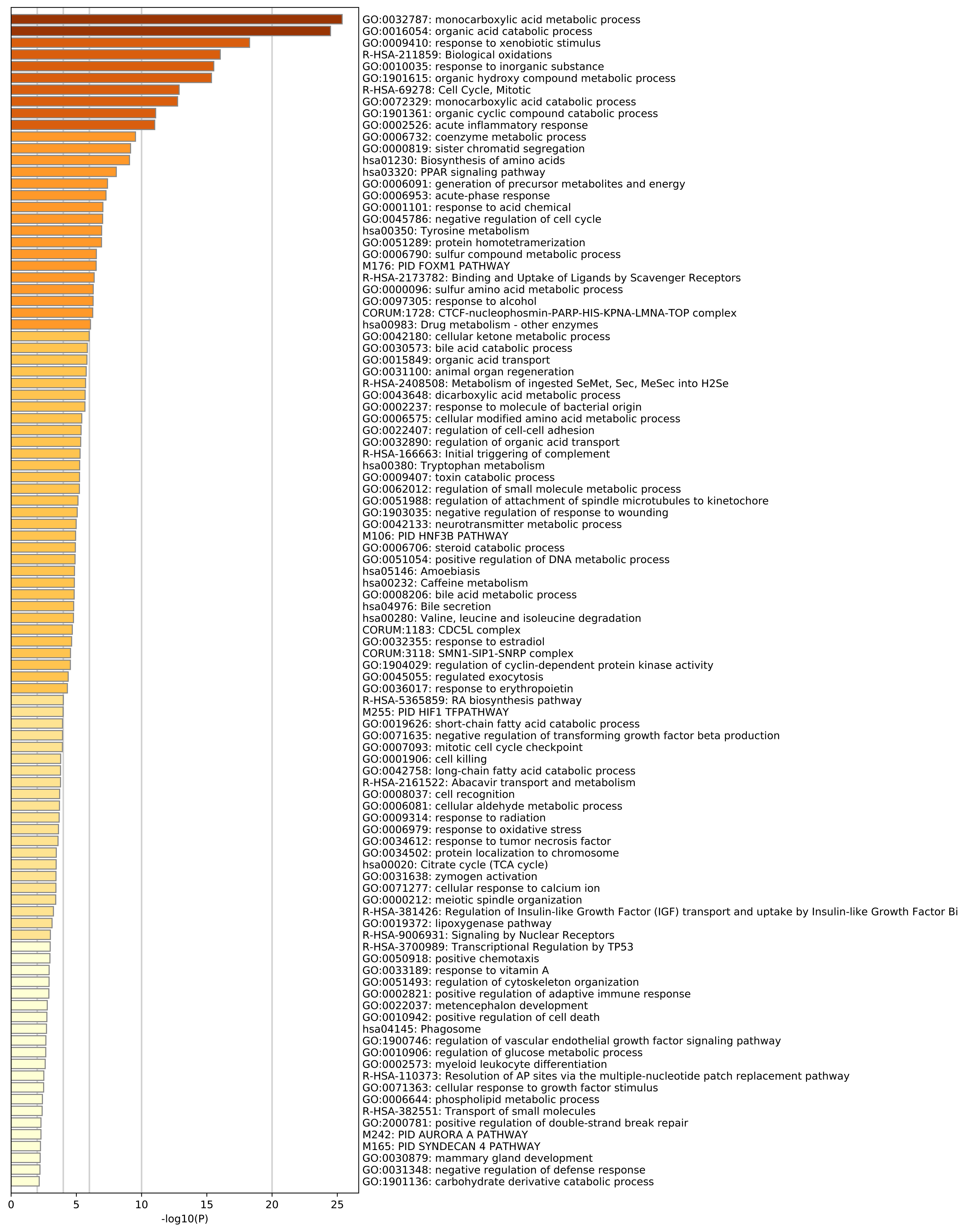

Supplement: Supplementary file 3 [file Image_2.JPEG]

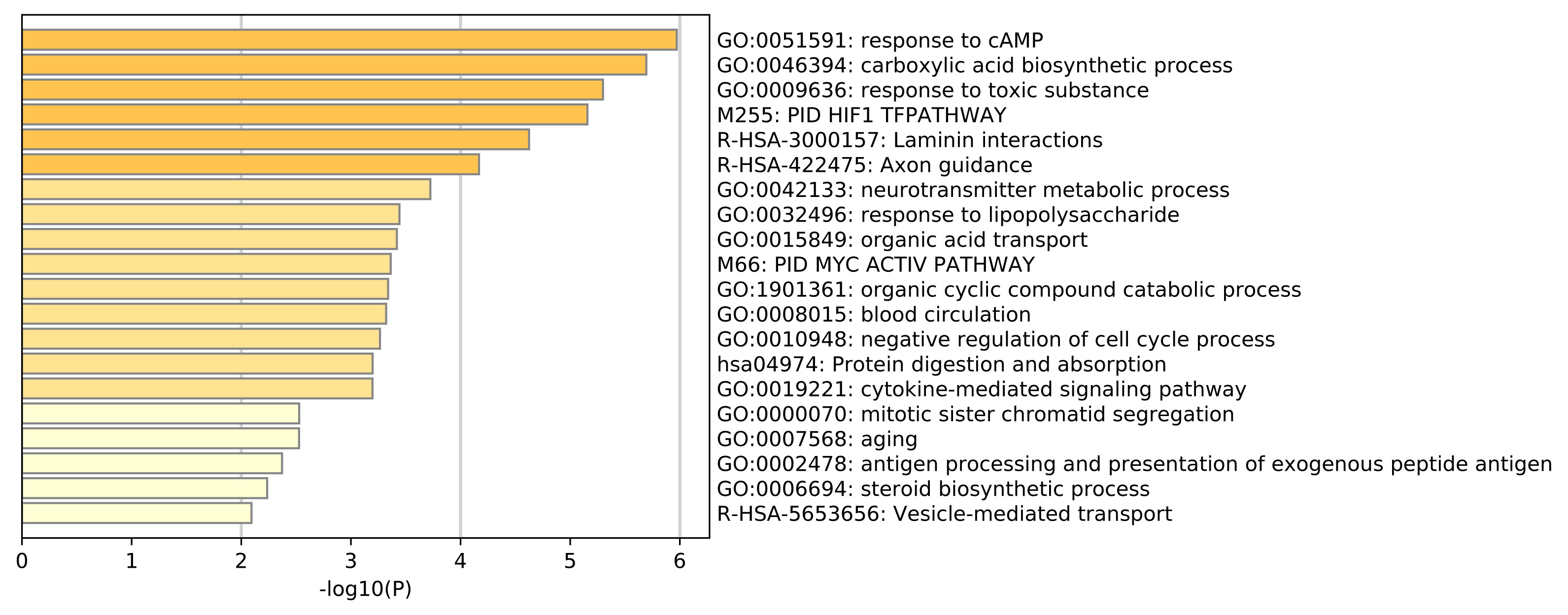

Supplement: Supplementary file 4 [file Image_3.JPEG]

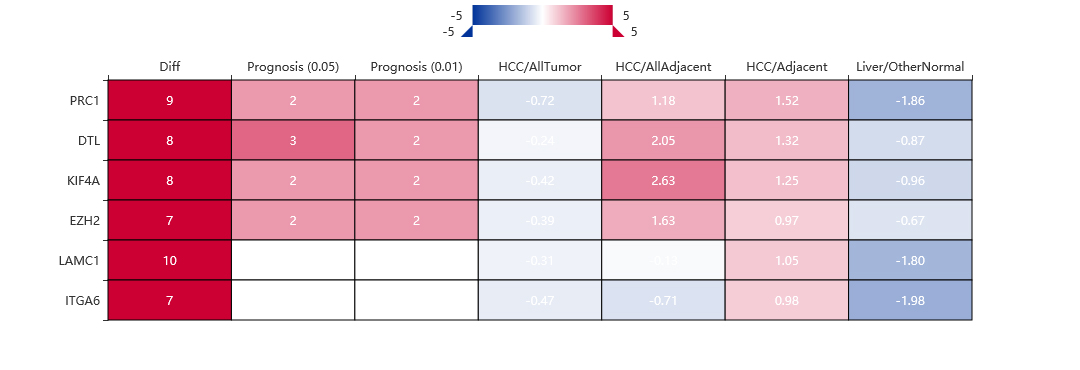

Supplement: Supplementary file 5 [file Image_4.JPEG]
